# Beyond *Aspergillus fumigatus*: The Clinical Burden of *Aspergillus flavus* and *Aspergillus niger* in Chronic Pulmonary Diseases

**DOI:** 10.3390/pathogens15060597

**Published:** 2026-06-01

**Authors:** Lisa Brizzolara, Jari Intra, Paola Faverio, Alice Biffi, Francesca Basta, Cristina Delfini, Nicoletta Novati, Elisa Zucchetti, Fabrizio Luppi, Marialuisa Lavitrano, Marco Casati

**Affiliations:** 1 Clinical Chemistry Laboratory, Fondazione IRCCS San Gerardo Dei Tintori, Via Pergolesi 33, 20900 Monza, Italy; l.brizzolara@campus.unimib.it (L.B.); francesca.basta@irccs-sangerardo.it (F.B.); cristina.delfini@irccs-sangerardo.it (C.D.); nicoletta.novati@irccs-sangerardo.it (N.N.); elisa.zucchetti@irccs-sangerardo.it (E.Z.); marco.casati@irccs-sangerardo.it (M.C.); 2 School of Medicine and Surgery, University of Milano-Bicocca, 20126 Milano, Italy; marialuisa.lavitrano@unimib.it; 3 Respiratory Diseases Unit, Fondazione IRCCS San Gerardo dei Tintori, 20900 Monza, Italy; paola.faverio@irccs-sangerardo.it (P.F.); alice.biffi@irccs-sangerardo.it (A.B.); fabrizio.luppi@unimib.it (F.L.)

**Keywords:** *Aspergillus fumigatus*, *Aspergillus* species, hypersensitivity pneumonitis, IgG antibodies, bronchiectasis, interstitial lung disease

## Abstract

**Background**: The clinical burden of pulmonary diseases associated with the genus *Aspergillus* is increasing, although diagnostic focus remains largely on *A. fumigatus*. This study evaluated the diagnostic value of testing for *A. flavus* and *A. niger*-specific IgG in patients with chronic respiratory conditions. **Methods**: A retrospective study was conducted on 274 subjects (156 with chronic respiratory diseases, Bronchiectasis, Hypersensitivity Pneumonitis [HP], and Interstitial Lung Disease [ILD] non-HP, and 67 healthy controls). Cut-off values were established at the 97.5th percentile of the control group: 30 mg/L for *A. fumigatus*, 13 mg/L for *A. flavus*, and 8 mg/L for *A. niger*. **Results**: Among 109 patients who tested negative for *A. fumigatus*, 49.5% showed positivity to at least one other species, preventing a significant diagnostic gap in exposure detection. Notably, 40.4% of these patients exhibited simultaneous positivity for both *A. flavus* and *A. niger*. In the HP subgroup, 100% of patients who were *A. fumigatus*-positive also showed concurrent positivity to the two other species. Furthermore, the Bronchiectasis group showed the highest rate of isolated *A. flavus* at 11.8%. **Conclusions**: Testing solely for *A. fumigatus* IgG leads to substantial underestimation of *Aspergillus* exposure. Integrating *A. flavus* and *A. niger* into diagnostic panels is essential for a comprehensive immunological assessment of fungal interaction, particularly in HP and ILD, where identifying specific antigenic exposure is crucial for managing chronic inflammation and preventing disease progression.

## 1. Introduction

The global burden of pulmonary diseases associated with the genus *Aspergillus* has reached critical levels, affecting millions of subjects with underlying respiratory conditions [1]. The genus comprises hundreds of species, but only a few are pathogenic. Among these, *A. fumigatus* is ubiquitous, yet the clinical relevance of *A. flavus* and *A. niger* is growing due to climate change and specific environmental exposures [2]. *Aspergillus* species are implicated in complex immune-mediated conditions. Hypersensitivity Pneumonitis (HP) is frequently linked to *Aspergillus* exposure and subsequent immune activation in damp indoor environments [3]. Furthermore, fungal presence complicates Bronchiectasis and Interstitial Lung Disease (ILD), driving a cycle of chronic inflammation and functional decline. International guidelines define *Aspergillus*-specific IgG as a cornerstone for identifying Chronic Pulmonary Aspergillosis (CPA) [4,5,6]. However, clinical presentations vary; *A. niger* is uniquely associated with pulmonary oxalosis [7], while *A. flavus* shows high virulence in specific geographic regions. The production of IgG antibodies against *Aspergillus* antigens represents either a chronic immune response to persistent fungal colonization, as seen in chronic non-allergic forms like CPA, or a Type III hypersensitivity reaction in allergic and immunologic conditions [3,4,5,6]. Unlike IgE, IgG titers reflect the total burden of antigen exposure. In patients with Bronchiectasis or ILD, the damaged lung architecture provides a niche for fungal persistence, leading to chronic stimulation of B-lymphocytes. In HP, these IgG antibodies act as “precipitins,” forming immune complexes with inhaled antigens that deposit in the alveolar walls, triggering fibrosis [8,9]. The immunological challenge lies in the shared epitopes between species. Proteins like Manganese Superoxide Dismutase (MnSOD) or heat shock proteins are highly conserved across *Aspergillus* spp., leading to significant serological cross-reactivity, which complicates the identification of the primary sensitizing agent [10]. This paper aims to define the diagnostic added value of testing for *A. flavus* and *A. niger* IgG, particularly in patients who test negative for *A. fumigatus*, and to analyze the implications of these species’ positivity in clinical practice.

## 2. Materials and Methods

### 2.1. Patients and Study Design

This retrospective study analyzed clinical and serological data retrieved from the database of the Clinical Laboratory at Fondazione IRCCS San Gerardo dei Tintori (Monza, Italy), spanning from 1 January 2022 to 31 December 2025. The study population consisted of 344 subjects, divided into three groups. Group I (*n* = 156) included patients with confirmed chronic respiratory conditions, further stratified into Bronchiectasis, Hypersensitivity Pneumonitis (HP), and Interstitial Lung Disease (ILD)-non HP. Group II (*n* = 67) comprised healthy individuals with no prior or subsequent diagnosis of pulmonary diseases for the determination of cut-off values. Group III (*n* = 70) named ‘Other Lung Pathology’ comprised patients with respiratory conditions who did not meet the specific criteria for bronchiectasis, HP, or non-HP ILD. This heterogeneous group included individuals diagnosed with asthma, Chronic Obstructive Pulmonary Disease (COPD), Asthma-COPD Overlap (ACO), and cystic fibrosis. Diagnostic confirmation was achieved through a multidisciplinary discussion approach, integrating clinical, radiological, and histopathological evidence. Specifically, the classification of HP adhered to the clinical guidelines (ATS/JRS/ALAT) for the diagnosis and management of HP developed by the American Thoracic Society (ATS), the Japanese Respiratory Society (JRS), and the Asociación Latinoamericana del Tórax (ALAT), and the guidelines released by the American College of Chest Physicians (CHEST). Only the first chronological result was included for subjects with multiple IgG determinations for the same antigen during the study period.

### 2.2. Determination of Specific IgG

Sera were collected by venous blood sampling and stored at 2–8 °C. Specific IgGs of the three antigens were quantified using a fluorescence enzyme immunoassay (FEIA) via ImmunoCAP technology, following the manufacturer’s recommendations (Thermo-Fisher Scientific, Phadia AB Uppsala, Uppsala, Sweden). There are no cut-off values defined by the manufacturer for each allergen. Concentrations are expressed in milligrams of IgG per liter (mg/L). The range recommended by the manufacturer is 2–200 mg/L, with a limit of detection of 2 mg/L. In cases of concentration values less than or greater than the limits of detection, they were reported as <2 and >200 mg/L, respectively, and no dilution was performed.

### 2.3. Statistical Analysis

All statistical calculations were performed using MedCalc for Windows, version 19.4 (MedCalc Software, Ostend, Belgium). Concentration values reported as <2 and >200 mg/L were assigned the values 2 and 200 mg/L, respectively. IgG results were expressed as median and range minimum/maximum values. Variables and differences in prevalence between the groups were analyzed using the Chi-squared (χ^2^) test. Statistical comparisons between the multiple disease subgroups were performed using the non-parametric Kruskal–Wallis test, followed by Dunn’s post hoc test with Bonferroni correction for pairwise comparisons. A *p*-value < 0.05 was considered statistically significant.

## 3. Results

The analysis of the study population revealed a predominantly elderly group across all categories. In the *A. fumigatus*-negative group, the median age was 71 years (Interquartile ranges (IQR), 66–77), with specific sub-groups showing median ages of 75 years (IQR, 62–77) for Bronchiectasis, 76 years (IQR, 67–78) for Hypersensitivity Pneumonitis (HP), and 73 years (IQR, 67–79) for non-HP Interstitial Lung Diseases (ILD). In the *A. fumigatus*-positive group, the median age was 67 years (IQR, 62–79), with 75 years (IQR, 66–79) for Bronchiectasis, 69 years (IQR, 67–76) for HP, and 70 years (IQR, 59–78) for non-HP ILD. Statistical comparison across the subgroups (Bronchiectasis, HP, and non-HP ILD) demonstrated that they were highly homogeneous, with no statistically significant differences in terms of median age, sex distribution, or the prevalence of baseline comorbidities (*p* > 0.05). In the preliminary phase of the study, non-parametric methods were used to determine cut-off values due to the differences between raw data and normal distribution, and the results were reported as medians and quantiles. The 97.5th percentile thresholds for *Aspergillus* spp. IgG was established by analyzing the healthy control group (*n* = 67), resulting in 30 mg/L for *A. fumigatus*, 13 mg/L for *A. flavus*, and 8 mg/L for *A. niger*.

The diagnostic accuracy and discriminative capacity of the non-parametrically established thresholds were subsequently analyzed using ROC curve analysis to determine the overall Area Under the Curve (AUC). Applying these cut-off values to the group of 109 patients with a confirmed pulmonary diagnosis who tested negative for *A. fumigatus* revealed a substantial diagnostic gap that would have resulted in a significant underestimation of fungal sensitization (Table 1). Within this *A. fumigatus*-negative population, only 50.4% of subjects (*n* = 55) were found to be seronegative for all tested species, resulting in a moderate diagnostic performance with AUC values ranging from 0.68 (95% Confidence Interval (CI): 0.61–0.75) to 0.77 (95% CI: 0.71–0.84). The cut-off values of 13 mg/L for *A. flavus* and 9 mg/L for *A. niger* yielded specificities of 72.2% (95% CI: 59.1–82.4%) and 75.9% (95% CI: 63.1–85.4%), respectively. Co-sensitization emerged as the dominant immunological trend, with 40.4% of patients (*n* = 44) exhibiting simultaneous positivity for both *A. flavus* and *A. niger*, while isolated reactivity to either species was notably infrequent, occurring in only 4.6% of cases (*n* = 5) for each species. Subgroup analysis further elucidated distinct clinical patterns: patients with Hypersensitivity Pneumonitis (HP) exhibited the most complex immune response, where double positivity reached 54.2%, resulting in a distinct *A. niger* sensitivity of 58.3% (95% CI: 38.8–75.5%) and an *A. flavus* sensitivity of 54.2% (95% CI: 35.1–72.1%), whereas only 4.2% showed isolated positivity for *A. niger*. In contrast, the Bronchiectasis group showed the highest rate of isolated *A. flavus* positivity at 11.8%, resulting in an *A. flavus* sensitivity of 52.9% (95% CI: 30.9–73.8%) and an *A. niger* sensitivity of 41.2% (95% CI: 21.6–63.9%). However, double positivity remained highly prevalent at 41.2%. In patients with non-HP interstitial lung diseases, while the total negativity rate remained higher at 54.4%, over one-third of the subjects (35.3%) demonstrated double positivity, despite lower individual sensitivities for *A. flavus* (39.7%) (95% CI: 28.9–51.6%) and *A. niger* (41.2%) (95%CI: 30.3–53.0%).

On the other hand, a completely distinct diagnostic pattern, marked by absolute triple positivity, was observed when evaluating the *A. fumigatus*-positive group. Concurrently, 100% of samples across all sub-stratifications, including Bronchiectasis, HP, and non-HP ILD, demonstrated concurrent reactivity toward both *A. flavus* and *A. niger*, yielding a 100.0% sensitivity across all diagnostic subgroups.

## 4. Discussion

The integration of species-specific IgG for *A. flavus* and *A. niger* fundamentally redefines the diagnostic approach to fungal-related lung diseases. Our most significant finding is that nearly half of the patients with chronic pulmonary conditions who test negative for *A. fumigatus* exhibit IgG positivity to *A. flavus* or *A. niger*. As previously noted by Ullah et al. [11], *A. flavus* can emerge as the dominant environmental pathogen in specific ecological niches; our data confirm that relying solely on *A. fumigatus* leads to significant underestimation of *Aspergillus* exposure. This is particularly critical in cases of HP and ILD, where the early detection of fungal triggers is vital to prevent irreversible disease progression [6]. These non-*fumigatus Aspergillus*-associated pulmonary exposure profiles represent a diagnostic challenge that necessitates broader serological screening [12]. The observation that 100% of *A. fumigatus*-positive patients also test positive for *A. flavus* and *A. niger* raises critical questions regarding cross-reactivity versus poly-fungal exposure. Many fungal antigens share high structural homology; historically, immunochemical analyses have shown that between 19% and 35% of antigenic fractions are shared among *A. fumigatus*, *A. flavus*, and *A. niger* [13]. Consequently, antibodies produced against *A. fumigatus* bind to similar conserved epitopes on other *Aspergillus* extracts [14,15]. Recent clinical evaluations confirm that significant cross-reactivity exists among different *Aspergillus* species [10]. Therefore, this overlapping serological signature most likely reflects extended humoral cross-reactivity to shared genus-specific antigens rather than true mixed environmental exposure for the entire cohort.

To capture this immune response accurately, contemporary immunoassays have demonstrated superior diagnostic sensitivity for detecting specific IgG compared to traditional immunoprecipitation methods [16]. Furthermore, recent evidence suggests that multiallergen screening tests, such as the mx4-IgE (a mixture including *A. fumigatus*, *A. alternata*, *A. niger*, and *A. cladosporioides*), demonstrated comparable or even superior diagnostic performance to species-specific IgE in identifying allergic bronchopulmonary aspergillosis (ABPA), reinforcing the clinical utility of genus-wide screening [17]. A patient exposed to one species is likely exposed to the entire genus, leading to a multi-species immune signature [18]. This serological profile suggests that once the threshold for the primary species is exceeded, the immune response consistently extends across the *Aspergillus* genus, likely due to high antigenic cross-reactivity or chronic poly-fungal environmental exposure. To better understand this balance between serological cross-reactivity and true mixed exposure, environmental and occupational histories were reviewed. Reliable exposure data were available for 135 out of 156 patients (86.5%). Subjects reported significant risk factors, including living in documented damp indoor environments or working in agricultural settings such as farms, where exposure to diverse mold species is amplified. Notably, 30 of these patients had also undergone collateral diagnostic testing, where true exposure and immunologic response were biologically corroborated by positive species-specific IgE against *A. flavus* or *A. niger* and/or positive galactomannan assays. These findings confirm that actual environmental co-exposure and specific immunological reactivity play a substantial role in a relevant subgroup of patients, rather than simple IgG cross-reactivity. Moreover, concerning the exposition of the 33 HP subjects, 25 (76%) were smokers, and 18 (54%) reported exposure to organic and chemical allergens due to the type of work, such as butcher, waiter, cook, mechanic, and painter, or to the presence of molds in the house.

Regarding the distribution of serological profiles, patients showing “triple positivity” (*A. fumigatus*, *A. flavus*, and *A. niger*) or double positivity, the characterization was based primarily on clinical data and serology. Laboratory isolation through fungal cultures was available only in a minority of cases (*n* = 5), as is frequent in retrospective studies where deep respiratory sampling is not always performed. Consequently, a positive IgG result represents a marker of chronic immune exposure rather than definitive mycological infection or active pathogenic colonization.

Antifungal treatments were administered to patients presenting with severe bronchiectasis or chronic phenotypes with high functional decline and multi-species seropositivity, where clinical and radiological criteria suggested active fungal disease. For other cases, particularly those with isolated non-*fumigatus* positivity, management focused predominantly on clinical monitoring, optimization of baseline therapies, and environmental remediation (such as addressing damp indoor triggers). Due to the retrospective nature, a definitive final clinical outcome is not available for all individuals, as several patients are still actively undergoing treatment. This lack of complete long-term outcome data precludes definitive conclusions regarding the comparative efficacy or final resolution rate of specific therapeutic interventions across the different serological subgroups.

Collectively, these data confirm that while *A. fumigatus* positivity serves as a definitive marker for genus-wide immune activation; its absence does not rule out clinically relevant exposure to other pathogenic *Aspergillus* species, particularly in patients suffering from HP and Bronchiectasis. As demonstrated by Pollock et al. [19] and Yang et al. [20], *Aspergillus* seropositivity correlates with increased exacerbation and hospitalization rates. Our results suggest that in ILD patients, the presence of “triple positivity” might reflect a higher total fungal burden or a more hyper-reactive immune system, both of which are associated with an accelerated reduction in lung function [6]. In HP patients, the identification of *A. niger* or *A. flavus* IgG, even in the absence of *A. fumigatus*, should immediately prompt an environmental investigation. In bronchiectasis, these findings support considering antifungal therapy to mitigate chronic inflammation, particularly when *Aspergillus*-related airway disease drives clinical deterioration [21].

Finally, regarding the underlying baseline etiology of patients with bronchiectasis, HP, and ILD, no significant differences were observed between the *Aspergillus*-positive and *Aspergillus*-negative subgroups. This lack of etiological divergence indicates that *Aspergillus* sensitization, particularly to non-*fumigatus* species like *A. flavus* and *A. niger*, occurs independently of the primary respiratory diagnosis. Consequently, the baseline etiology alone cannot predict which patients will develop non-*fumigatus* positivity, further underscoring the clinical necessity of expanded IgG screening panels across all patients with chronic lung diseases to identify hidden fungal complications that share identical clinical presentations.

Some limitations of this study must be acknowledged: (1) the work was primarily limited by its single-center retrospective design, which may restrict the generalizability of findings to different geographical regions; (2) the high antigenic cross-reactivity among Aspergillus species represents a major confounding factor, making it difficult to differentiate true poly-fungal exposure from shared epitope recognition in the majority of patients; (3) the lack of routine microbiological and radiological correlation in all seropositive patients prevents us from establishing a direct causative role of non-*fumigatus* species in active disease progression.

## 5. Conclusions

Collectively, expanding IgG panels to include *A. flavus* and *A. niger* is essential to eliminate the diagnostic gap caused by relying solely on *A. fumigatus*. Our findings underlined that species-specific testing identifies previously unrecognized profiles of fungal exposure, particularly in HP and ILD cases. Implementing these expanded screenings demonstrates potential diagnostic utility for a more comprehensive exposure assessment, mitigating chronic inflammation and preventing irreversible functional lung decline in patients with chronic respiratory diseases. Prospective studies are required to confirm the clinical utility and prognostic value of expanded IgG screening.

## Figures and Tables

**Table 1:** Distribution of *Aspergillus* positivity in the population study.

Diagnosis		*Aspergillus fumigatus* (Negative)				
		Samples (*n*)	*A. flavus* (Positive) *n* (%)	*A. niger* (Positive) *n* (%)	*A. flavus* and *niger* (Positive) *n* (%)	*A. flavus* and *niger* (negative) *n* (%)
Lung pathology		109	5 (4.6)	5 (4.6)	44 (40.4)	55 (50.4)
	Bronchiectasis	17	2 (11.8)	0 (0.0)	7 (41.2)	8 (47.0)
	HP	24	0 (0.0)	1 (4.3)	13 (54.2)	10 (41.6)
	ILD (non-HP)	68	3 (4.4)	4 (5.9)	24 (35.3)	37 (54.4)
Other Lung pathology		54	5 (9.2)	3 (5.5)	10 (18.6)	36 (66.7)
		***Aspergillus fumigatus* (Positive)**				
		Samples (*n*)	*A. flavus* (Positive) *n* (%)	*A. niger* (Positive) *n* (%)	*A. flavus and niger* (Positive) *n* (%)	*A. flavus and niger* (negative) *n* (%)
Lung pathology		47	0 (0.0)	0 (0.0)	47 (100.0)	0 (0.0)
	Bronchiectasis	10	0 (0.0)	0 (0.0)	10 (100.0)	0 (0.0)
	HP	9	0 (0.0)	0 (0.0)	9 (100.0)	0 (0.0)
	ILD (non-HP)	28	0 (0.0)	0 (0.0)	28 (100.0)	0 (0.0)
Other Lung pathology		16	0 (0.0)	0 (0.0)	15 (93.7)	1 (6.3)

## Data Availability

Data presented in this study are available on request from the corresponding author.
